# Tri­methyl­phosphine oxide dihydrate

**DOI:** 10.1107/S2414314623003140

**Published:** 2023-04-14

**Authors:** Matic Urlep, Janez Cerkovnik, Matic Lozinšek

**Affiliations:** a Department of Chemistry and Biochemistry, Faculty of Chemistry and Chemical Technology, University of Ljubljana, Večna pot 113, 1000 Ljubljana, Slovenia; b Jožef Stefan Institute, Jamova cesta 39, 1000 Ljubljana, Slovenia; University of Aberdeen, United Kingdom

**Keywords:** phosphine oxide, hydrate, hydrogen bonding, crystal structure

## Abstract

The title hydrate, Me_3_PO·2H_2_O, crystallizes in the ortho­rhom­bic space group *Pbca* with eight formula units per unit cell. The extended structure displays O—H⋯O hydrogen bonding, with Me_3_PO mol­ecules as acceptors and water mol­ecules acting as donors and acceptors of hydrogen bonds, forming hydrogen-bonded layers, which propagate in the *ac* plane.

## Abstract 2

Tri­methyl­phosphine oxide and water mol­ecules form 16-membered hydrogen-bonded rings inter­connected into layers.

## Graphical abstract

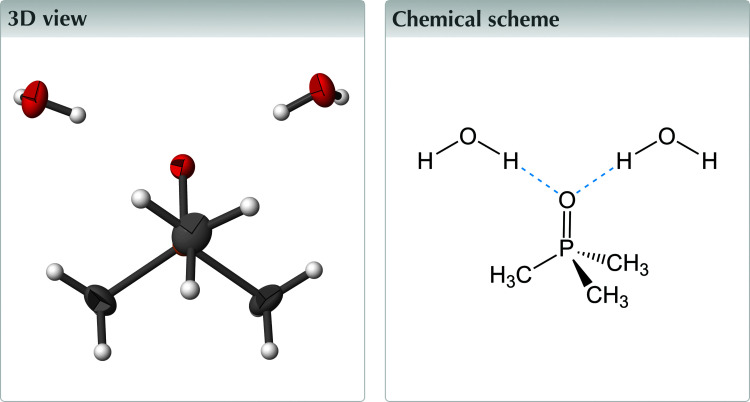

## Structure description

Tertiary phosphine oxides, *R*_3_P=O (*R* = alk­yl/ar­yl), are good hydrogen-bond acceptors and have been employed for co-crystallization and stabilization of hydrogen-bond donor species such as hydrogen peroxide (Arp *et al.*, 2019 ▸) and di(hydro­per­oxy)alkanes (Ahn *et al.*, 2015 ▸). However, only a limited number of simple tertiary phosphine oxide hydrates have been structurally characterized by single-crystal X-ray diffraction. For example: tri­cyclo­hexyl­phosphine oxide hydrate, Cy_3_PO·H_2_O (Cambridge Structural Database refcode ZOQHEU; Hilliard *et al.* 2014 ▸; Thomas *et al.*, 2019 ▸); tri­phenyl­phosphine oxide hemihydrate, Ph_3_PO·0.5H_2_O (JEDTOB; Baures & Silverton, 1990 ▸; Baures, 1991 ▸; Ng, 2009 ▸); tri-*p*-tolyl­phosphine oxide hemihydrate *p*-Tol_3_PO·0.5H_2_O (JULBAT; Churchill *et al.*, 1993 ▸); tris­(2,4,6-tri­meth­oxy­phen­yl)phosphine oxide hydrate, [(CH_3_O)_3_C_6_H_2_]_3_PO·H_2_O (WAMXIR; Chaloner *et al.*, 1993 ▸); tris­(2,4,6-tri­meth­oxy­phen­yl)phosphine oxide dihydrate, [(CH_3_O)_3_C_6_H_2_]_3_PO·2H_2_O (LICVUO; Dunbar & Haefner, 1994 ▸); di-*o*-tolyl­phenyl­phosphine oxide hydrate, *o*-Tol_2_PhPO·H_2_O (POMRUH; Arp *et al.*, 2019 ▸). The absence of crystal structures of tri­alkyl­phosphine oxide hydrates with a short alkyl chain is particularly noteworthy. Herein, the crystal structure of the title phosphine oxide hydrate is reported.

**Figure 1:**
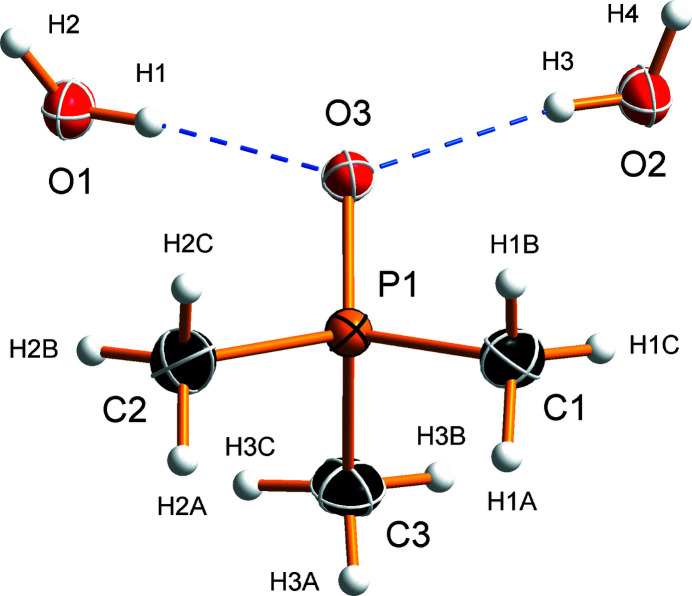
The asymmetric unit and the atom-labelling scheme of the Me_3_PO·2H_2_O crystal structure. Displacement ellipsoids are depicted at the 50% probability level, hydrogen atoms are shown as spheres of arbitrary radius, and hydrogen bonds are indicated by blue dashed lines.

Tri­methyl­phosphine oxide dihydrate crystallizes in the ortho­rhom­bic space group *Pbca* with one Me_3_PO and two H_2_O mol­ecules in the asymmetric unit (Fig. 1 ▸). The P=O bond length [1.5067 (7) Å] and P—C distances [1.7805 (12), 1.7809 (11), and 1.7819 (11) Å] are in good agreement with the bond distances reported in crystal structures of tri­methyl­phosphine oxide (FAKLUY; Engelhardt *et al.*, 1986 ▸; Begimova *et al.*, 2016 ▸).

**Figure 2:**
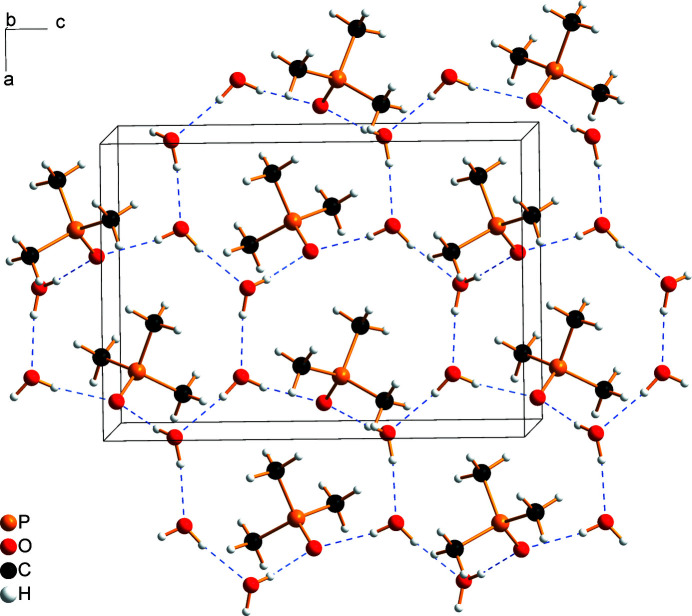
Hydrogen-bonded rings (depicted by blue dashed lines) are conjoined into layers parallel to the *ac*-plane.

**Figure 3:**
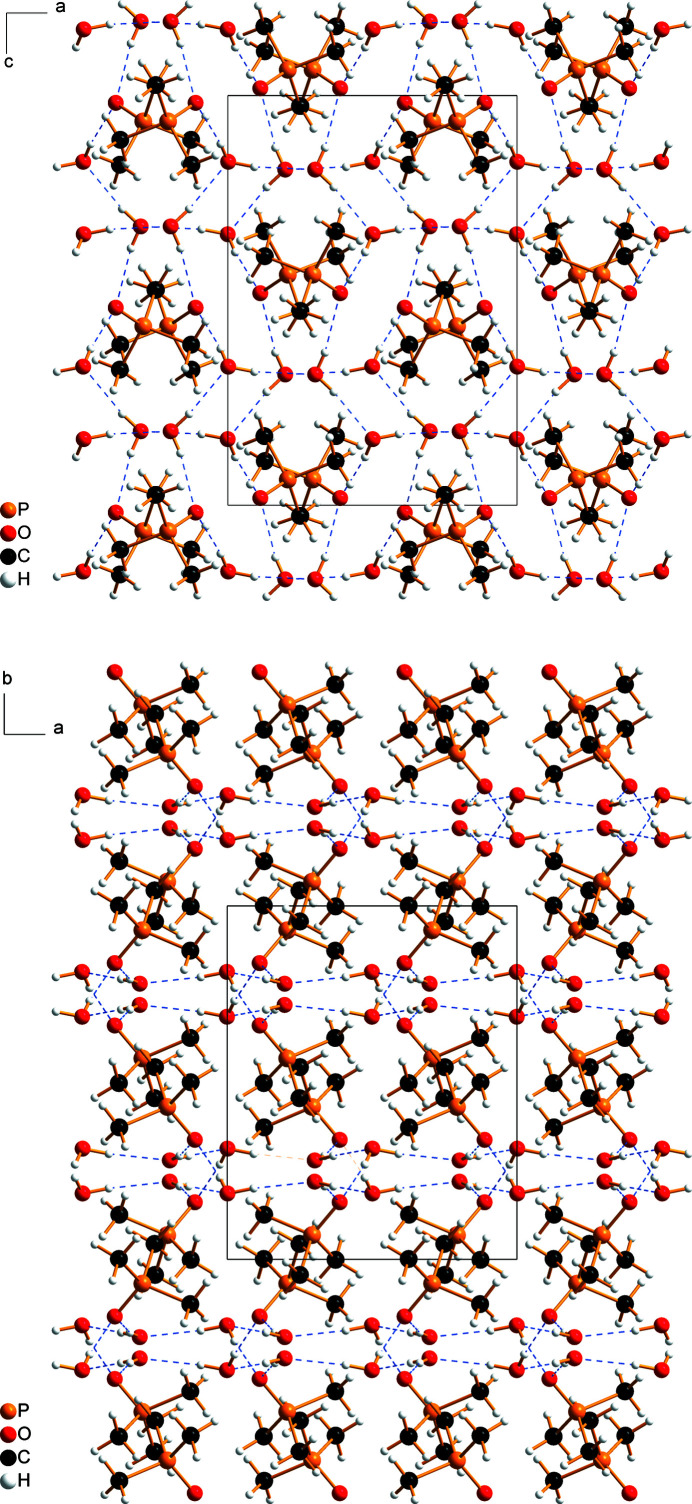
Crystal packing and the unit cell of Me_3_PO·2H_2_O viewed along the crystallographic *b*-axis (top) and *c*-axis (bottom). Hydrogen bonds are indicated by blue dashed lines.

The tri­methyl­phosphine oxide mol­ecule is an acceptor of two O⋯H—O hydrogen bonds, whereas both water mol­ecules are donors of hydrogen bonds to Me_3_PO and H_2_O, and acceptors of hydrogen bonds from adjacent water mol­ecules (Table 1 ▸, Fig. 2 ▸). Two Me_3_PO and six H_2_O mol­ecules form a hydrogen-bonded 16-membered ring (Fig. 2 ▸) with an 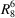 (16) graph-set motif (Etter, 1990 ▸). Each water mol­ecule participates in three rings, whereas the tri­methyl­phosphine mol­ecule participates in two rings. These rings are inter­connected into layers that extend parallel to the *ac* plane, whereby each ring is surrounded by six other rings (Figs. 2 ▸, 3 ▸). Hydrogen-bonded layers and layers of Me_3_P groups are stacked along the *b*-axis direction (Fig. 3 ▸).

## Synthesis and crystallization

Tri­methyl­phosphine oxide (2.3 mg) was dissolved in a mixture of acetone-*d*_6_ (0.6 ml) and diethyl ether-*d*_10_ (0.3 ml) in an NMR tube that was capped and cooled to −20 °C in an ethanol cooling bath. The Dewar flask containing the bath and sample was sealed and placed in a freezer at −80 °C. Crystals of Me_3_PO·2H_2_O grew within 3 days. The single crystals were examined, selected, and transferred to the diffractometer employing a previously described low-temperature crystal-mounting procedure (Lozinšek *et al.*, 2021 ▸). The crystals melt at room temperature.

## Refinement

Crystal data, data collection, and structure refinement details are summarized in Table 2 ▸. Positions and isotropic thermal displacement parameters of hydrogen atoms were freely refined (Cooper *et al.*, 2010 ▸).

## Supplementary Material

Crystal structure: contains datablock(s) I. DOI: 10.1107/S2414314623003140/hb4428sup1.cif

Structure factors: contains datablock(s) I. DOI: 10.1107/S2414314623003140/hb4428Isup2.hkl

Supporting information file. DOI: 10.1107/S2414314623003140/hb4428Isup3.cml

CCDC reference: 2254008

Additional supporting information:  crystallographic information; 3D view; checkCIF report

## Figures and Tables

**Table 1:** Hydrogen-bond geometry (Å, °)

*D*—H⋯*A*	*D*—H	H⋯*A*	*D*⋯*A*	*D*—H⋯*A*
O1—H1⋯O3	0.807 (17)	1.976 (18)	2.7787 (10)	173.2 (16)
O2—H3⋯O3	0.789 (18)	2.046 (18)	2.8261 (11)	169.7 (15)
O1—H2⋯O2^i^	0.873 (18)	1.981 (18)	2.8460 (12)	170.6 (14)
O2—H4⋯O1^ii^	0.834 (17)	1.993 (17)	2.8218 (11)	172.0 (14)

Symmetry codes: (i) 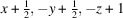 ; (ii) 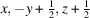 .

**Table 2:** Experimental details

Crystal data	
Chemical formula	C_3_H_9_OP·2H_2_O
*M*_r_	128.10
Crystal system, space group	Orthorhombic, *P* *b* *c* *a*
Temperature (K)	150
*a*, *b*, *c* (Å)	9.33514 (8), 11.39118 (9), 13.23961 (11)
*V* (Å^3^)	1407.88 (2)
*Z*	8
Radiation type	Cu *K*α
μ (mm^−1^)	2.88
Crystal size (mm)	0.70 × 0.24 × 0.13

Data collection	
Diffractometer	SuperNova, Dual, Cu at home/near, Atlas
Absorption correction	Gaussian (*CrysAlis PRO*; Rigaku OD, 2023 ▸)
*T*_min_, *T*_max_	0.299, 1.000
No. of measured, independent and observed [*I* > 2σ(*I*)] reflections	24977, 1451, 1417
*R*_int_	0.023
(sin θ/λ)_max_ (Å^−1^)	0.628

Refinement	
*R*[*F* ^2^ > 2σ(*F* ^2^)], *wR*(*F* ^2^), *S*	0.021, 0.057, 1.04
No. of reflections	1451
No. of parameters	117
H-atom treatment	All H-atom parameters refined
Δρ_max_, Δρ_min_ (e Å^−3^)	0.27, −0.24

Computer programs: *CrysAlis PRO* (Rigaku OD, 2023 ▸), *SHELXT* (Sheldrick, 2015*a* ▸), *SHELXL* (Sheldrick, 2015*b* ▸), *OLEX2* (Dolomanov *et al.*, 2009 ▸), *DIAMOND* (Brandenburg, 2005 ▸) and *publCIF* (Westrip, 2010 ▸).

## full crystallographic data

### Crystal data

**Table:**

C_3_H_9_OP·2H_2_O	*D*_x_ = 1.209 Mg m^−^^3^
*M**_r_* = 128.10	Cu *K*α radiation, λ = 1.54184 Å
Orthorhombic, *P**b**c**a*	Cell parameters from 18569 reflections
*a* = 9.33514 (8) Å	θ = 3.3–75.4°
*b* = 11.39118 (9) Å	µ = 2.88 mm^−^^1^
*c* = 13.23961 (11) Å	*T* = 150 K
*V* = 1407.88 (2) Å^3^	Block, colourless
*Z* = 8	0.70 × 0.24 × 0.13 mm
*F*(000) = 560	

### Data collection

**Table:**

SuperNova, Dual, Cu at home/near, Atlas diffractometer	1451 independent reflections
Radiation source: micro-focus sealed X-ray tube, SuperNova (Cu) X-ray Source	1417 reflections with *I* > 2σ(*I*)
Mirror monochromator	*R*_int_ = 0.023
Detector resolution: 5.2466 pixels mm^-1^	θ_max_ = 75.5°, θ_min_ = 7.0°
ω scans	*h* = −11→11
Absorption correction: gaussian (CrysalisPro; Rigaku OD, 2023)	*k* = −14→14
*T*_min_ = 0.299, *T*_max_ = 1.000	*l* = −16→16
24977 measured reflections	

### Refinement

**Table:**

Refinement on *F*^2^	Hydrogen site location: difference Fourier map
Least-squares matrix: full	All H-atom parameters refined
*R*[*F*^2^ > 2σ(*F*^2^)] = 0.021	*w* = 1/[σ^2^(*F*_o_^2^) + (0.032*P*)^2^ + 0.4017*P*] where *P* = (*F*_o_^2^ + 2*F*_c_^2^)/3
*wR*(*F*^2^) = 0.057	(Δ/σ)_max_ = 0.001
*S* = 1.04	Δρ_max_ = 0.27 e Å^−^^3^
1451 reflections	Δρ_min_ = −0.24 e Å^−^^3^
117 parameters	Extinction correction: *SHELXL2019/2* (Sheldrick, 2015b), Fc^*^=kFc[1+0.001xFc^2^λ^3^/sin(2θ)]^-1/4^
0 restraints	Extinction coefficient: 0.0019 (4)
Primary atom site location: dual	

### Special details

**Table:**

Geometry. All esds (except the esd in the dihedral angle between two l.s. planes) are estimated using the full covariance matrix. The cell esds are taken into account individually in the estimation of esds in distances, angles and torsion angles; correlations between esds in cell parameters are only used when they are defined by crystal symmetry. An approximate (isotropic) treatment of cell esds is used for estimating esds involving l.s. planes.

### Fractional atomic coordinates and isotropic or equivalent isotropic displacement parameters (Å^2^)

**Table:**

	*x*	*y*	*z*	*U*_iso_*/*U*_eq_	
P1	0.29293 (2)	0.43133 (2)	0.43709 (2)	0.01874 (11)	
O3	0.38810 (7)	0.33831 (6)	0.48229 (5)	0.02332 (17)	
O1	0.50092 (9)	0.18661 (7)	0.33846 (6)	0.02949 (19)	
O2	0.30184 (8)	0.28005 (8)	0.68067 (6)	0.0309 (2)	
C3	0.12748 (12)	0.37348 (12)	0.39205 (10)	0.0361 (3)	
C1	0.25087 (15)	0.54386 (11)	0.52559 (9)	0.0360 (3)	
C2	0.37390 (13)	0.50121 (11)	0.33118 (9)	0.0363 (3)	
H1	0.4749 (17)	0.2314 (14)	0.3820 (13)	0.049 (4)*	
H3	0.3357 (17)	0.2929 (13)	0.6272 (13)	0.044 (4)*	
H1A	0.1928 (16)	0.6023 (14)	0.4936 (12)	0.049 (4)*	
H3A	0.0705 (17)	0.4349 (13)	0.3682 (13)	0.049 (4)*	
H2	0.5911 (19)	0.2011 (13)	0.3261 (11)	0.048 (4)*	
H3B	0.0772 (19)	0.3342 (15)	0.4489 (14)	0.062 (5)*	
H4	0.3659 (17)	0.2931 (12)	0.7231 (12)	0.042 (4)*	
H2A	0.3129 (17)	0.5592 (14)	0.3018 (13)	0.050 (4)*	
H3C	0.148 (2)	0.3195 (15)	0.3342 (15)	0.068 (5)*	
H2B	0.395 (2)	0.4400 (15)	0.2808 (14)	0.063 (5)*	
H1C	0.2035 (16)	0.5079 (16)	0.5804 (12)	0.055 (5)*	
H2C	0.457 (2)	0.5422 (15)	0.3541 (14)	0.066 (5)*	
H1B	0.337 (2)	0.5830 (16)	0.5464 (14)	0.067 (5)*	

### Atomic displacement parameters (Å^2^)

**Table:**

	*U*^11^	*U*^22^	*U*^33^	*U*^12^	*U*^13^	*U*^23^
P1	0.01726 (15)	0.02106 (16)	0.01790 (16)	0.00154 (8)	0.00075 (7)	0.00121 (8)
O3	0.0247 (3)	0.0246 (3)	0.0206 (3)	0.0062 (3)	0.0018 (2)	0.0022 (3)
O1	0.0265 (4)	0.0370 (4)	0.0249 (4)	0.0048 (3)	0.0004 (3)	−0.0075 (3)
O2	0.0240 (4)	0.0472 (5)	0.0214 (4)	−0.0027 (3)	0.0013 (3)	0.0014 (3)
C3	0.0252 (5)	0.0464 (7)	0.0366 (6)	−0.0071 (5)	−0.0062 (5)	0.0026 (5)
C1	0.0422 (7)	0.0322 (5)	0.0336 (6)	0.0156 (5)	−0.0049 (6)	−0.0071 (5)
C2	0.0336 (6)	0.0393 (6)	0.0359 (6)	0.0022 (5)	0.0068 (5)	0.0174 (5)

### Geometric parameters (Å, º)

**Table:**

P1—O3	1.5067 (7)	C3—H3B	0.993 (18)
P1—C3	1.7819 (11)	C3—H3C	1.001 (19)
P1—C1	1.7805 (12)	C1—H1A	0.957 (17)
P1—C2	1.7809 (11)	C1—H1C	0.943 (17)
O1—H1	0.807 (17)	C1—H1B	0.96 (2)
O1—H2	0.873 (18)	C2—H2A	0.955 (17)
O2—H3	0.789 (18)	C2—H2B	0.985 (18)
O2—H4	0.834 (17)	C2—H2C	0.958 (19)
C3—H3A	0.934 (16)		
			
O3—P1—C3	112.58 (5)	H3B—C3—H3C	113.3 (13)
O3—P1—C1	112.02 (5)	P1—C1—H1A	109.6 (10)
O3—P1—C2	112.13 (5)	P1—C1—H1C	107.3 (11)
C1—P1—C3	107.18 (7)	P1—C1—H1B	109.7 (11)
C1—P1—C2	106.85 (7)	H1A—C1—H1C	112.1 (13)
C2—P1—C3	105.64 (6)	H1A—C1—H1B	106.2 (14)
H1—O1—H2	107.7 (14)	H1C—C1—H1B	112.0 (15)
H3—O2—H4	106.5 (15)	P1—C2—H2A	112.1 (10)
P1—C3—H3A	109.3 (9)	P1—C2—H2B	107.6 (10)
P1—C3—H3B	108.8 (10)	P1—C2—H2C	108.3 (11)
P1—C3—H3C	108.3 (11)	H2A—C2—H2B	109.5 (14)
H3A—C3—H3B	108.9 (14)	H2A—C2—H2C	106.1 (13)
H3A—C3—H3C	108.2 (14)	H2B—C2—H2C	113.3 (15)

### Hydrogen-bond geometry (Å, º)

**Table:**

*D*—H···*A*	*D*—H	H···*A*	*D*···*A*	*D*—H···*A*
O1—H1···O3	0.807 (17)	1.976 (18)	2.7787 (10)	173.2 (16)
O2—H3···O3	0.789 (18)	2.046 (18)	2.8261 (11)	169.7 (15)
O1—H2···O2^i^	0.873 (18)	1.981 (18)	2.8460 (12)	170.6 (14)
O2—H4···O1^ii^	0.834 (17)	1.993 (17)	2.8218 (11)	172.0 (14)

Symmetry codes: (i) *x*+1/2, −*y*+1/2, −*z*+1; (ii) *x*, −*y*+1/2, *z*+1/2.
